# The N-terminal ELR^+^ motif of the neutrophil attractant CXCL8 confers susceptibility to degradation by the Group A streptococcal protease, SpyCEP

**DOI:** 10.1016/j.jbc.2025.108448

**Published:** 2025-03-25

**Authors:** Sean Patrick Giblin, Sophie McKenna, Stephen Matthews, Shiranee Sriskandan, James Edward Pease

**Affiliations:** 1 National Heart and Lung Institute, Imperial College London, London, United Kingdom; 2 Department of Life Sciences, Imperial College London, London, United Kingdom; 3 Centre for Bacterial Resistance Biology, Imperial College London, London, United Kingdom; 4 Department of Infectious Disease, Imperial College London, London, United Kingdom

**Keywords:** chemokine, CXCL8, enzyme degradation, protease, recombinant protein expression, SpyCEP, *Streptococcus pyogenes* (*S. pyogenes*)

## Abstract

*Streptococcus pyogenes* (Group A *Streptococcus* or GAS) is a major human pathogen for which an effective vaccine is highly desirable. Invasive *S. pyogenes* strains evade the host immune response in part by producing a cell envelope protease, SpyCEP. This neutralizes chemokines containing an N-terminal Glu-Leu-Arg motif (ELR^+^ chemokines) by cleavage at a distal C-terminal site within the chemokine. SpyCEP is a component of several *S. pyogenes* vaccines, yet the molecular determinants underlying substrate selectivity are poorly understood. We hypothesized that chemokine recognition and cleavage is a multistep process involving distinct domains of both substrate and enzyme. We generated a panel of recombinant CXCL8 variants where domains of the chemokine were exchanged or mutated. Chemokine degradation by SpyCEP was assessed by SDS-PAGE, Western blot, and ELISA. Extension of the CXCL8 N-terminus was found to inhibit chemokine cleavage. Reciprocal exchanges of the N-termini of CXCL8 with that of the ELR^-^ chemokine CXCL4 resulted in the generation of loss of function and gain of function substrates. This suggested a key role for the ELR motif in substrate recognition, which was supported directly by alanine substitution of the ELR motif of CXCL8, impairing the parameters, K_M_, V_max_, and Kcat in kinetic assays with SpyCEP. Collectively, our findings identify the N-terminal ELR motif as a major determinant for recognition by SpyCEP and expose a vulnerability in the mechanism by which the protease recognises its substrates. This likely presents potential avenues for therapeutic intervention *via* targeted vaccine design and small molecule inhibition.

*Streptococcus pyogenes* (Group A *Streptococcus* or GAS) infections are restricted to humans, with outcomes ranging from pharyngitis and scarlet fever to life-threatening necrotizing soft-tissue infections (NSTI) (1). Neutrophils are the first immune cells to respond to GAS infections and are specifically recruited to sites of bacterial infection by chemoattractants acting on cell-surface receptors (2), notably the CXC subset of chemokines of which CXCL8 is a principal member (3). NSTI are associated with the increased expression of the cell surface envelope protease SpyCEP (4) which cleaves the C-terminus of several neutrophil-recruiting CXC chemokines, significantly reducing their potency, and effecting evasion of the host immune response (5). Current treatments for invasive *S. pyogenes* infection predominantly rely upon the sensitivity of the bacteria to β-lactams, where emergence of antibiotic resistance remains a threat (6); indeed, *S. pyogenes* is now in the World Health Organization’s list of priority pathogens due to the risk of emerging macrolide resistance (7). To support vaccine development against *S.pyogenes*, the WHO published a technology road map (8, 9), with SpyCEP being an antigen included in at least four combination vaccines currently in clinical or pre-clinical trials (10, 11, 12, 13), reviewed in (14, 15).

Immunization of mice with a catalytically dead form of the full-length SpyCEP protein has been shown to induce an antibody response, with anti-sera able to impair *in vitro* cleavage of the chemokine CXCL8 by SpyCEP and offer significant protection following intranasal infection with SpyCEP-expressing *S. pyogenes* (16). What remains unknown are the epitopes within SpyCEP against which the most productive neutralizing antibody responses might be generated. This is closely linked to a lack of understanding of how SpyCEP interacts with substrates, despite access to high-resolution SpyCEP structures (16, 17).

Previous research has highlighted the ability of SpyCEP to inactivate the CXC chemokines CXCL1, CXCL2, CXCL3, CXCL5, CXCL6, CXCL7 and CXCL8, whilst other CXC chemokines such as CXCL4, CXCL9, CXCL10, CXCL11 and CXCL12 are resistant to cleavage and inactivation (5, 18). We generated recombinant variants of CXCL8 to probe the mechanism by which chemokines are recognized and cleaved by SpyCEP. We experimentally confirm that the N-terminal ELR motif of CXCL8 is a key determinant of substrate recognition by SpyCEP and propose a multi-step model of catalysis in which binding of the chemokine N-terminal ELR motif, precedes cleavage at the distal C-terminus.

## Results

**Figure 1:**
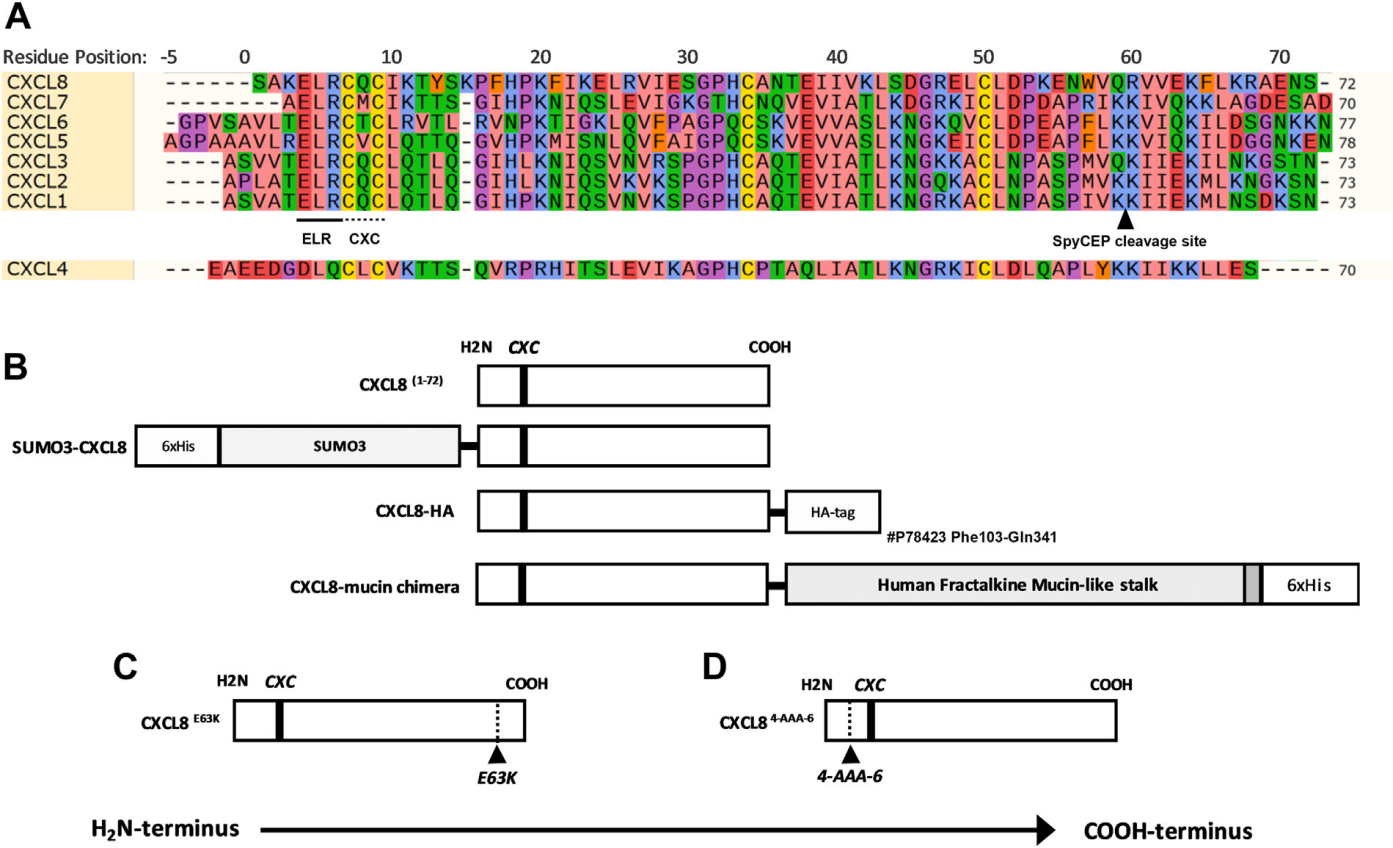
**Recombinant CXCL8 forms used in the study.***A*, alignment of mature CXC chemokines relevant to the study. Numbering is relative to the CXCL8 sequence. The ELR motif, CXC motif, and SpyCEP cleavage site are indicated. *B-D*, schematic representation of recombinant CXCL8 species generated for use in this study: (B) WT CXCL8 (1–72), SUMO3-CXCL8, CXCL8-HA and CXCL8-mucin stalk chimera. *C*, CXCL8 E63K mutant. *D*, CXCL8 ^4-AAA-6^ mutant.

SpyCEP preferentially cleaves a subset of CXC chemokines with an N-terminal ELR motif which are known collectively as ELR^+^ chemokines, exemplified by CXCL8 (Fig. 1*A*). ELR^-^ CXC chemokines (typified by CXCL4) are not SpyCEP substrates. We therefore hypothesized that the ELR motif is a key binding determinant for SpyCEP substrates and tested this by a program of mutagenesis. We generated N- and C-terminally extended forms of CXCL8 (Fig. 1*B*), and a C-terminal point mutation, CXCL8 E63K (Fig. 1*C*). An ELR-deficient CXCL8 mutant, where the ELR motif was replaced by alanine (CXCL8 ^4-AAA-6^), was also generated (Fig. 1*D*). Recombinant chemokines were produced in *E. coli* using established methodologies (19).

### N-terminal extension of CXCL8 protects it from cleavage by SpyCEP

**Figure 2:**
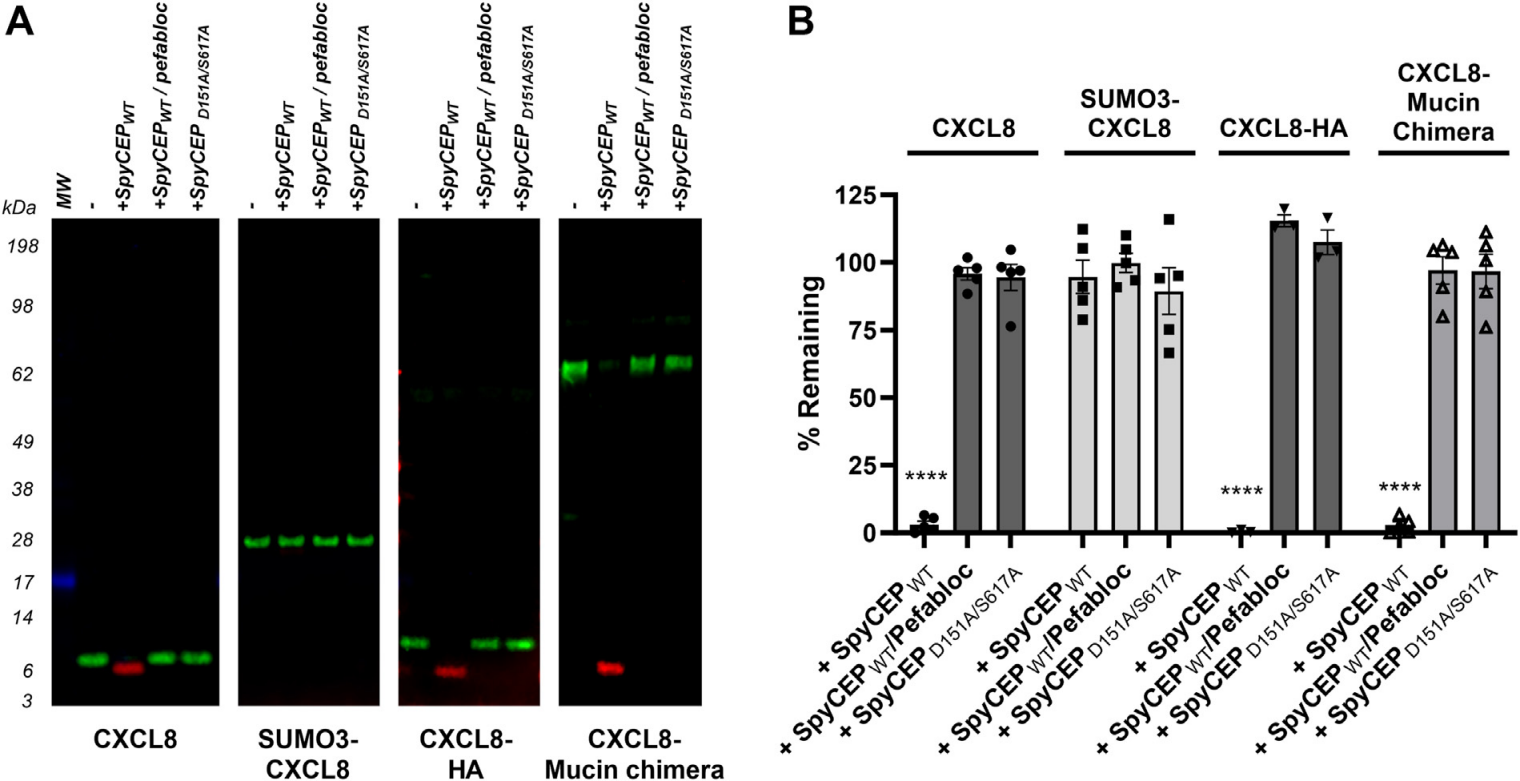
**SpyCEP tolerates C-terminal but not N-terminal extensions of CXCL8.***A*, following incubation of recombinant chemokines with SpyCEP for 16 h, products were imaged by multiplex Western blot. Data are representative of 3 separate experiments. *B*, quantification of chemokine cleavage as determined by ELISA (n = 5).

We assessed the ability of SpyCEP to cleave N- and C-terminally extended forms of CXCL8. Recombinant chemokines were incubated with either SpyCEP, SpyCEP pretreated with the inhibitor pefabloc (20), or catalytically inactive SpyCEP D151A/S617A (16) (Fig. 2*A*). Following incubation, reaction products were resolved by SDS-PAGE and probed by 2-color multiplex Western blot (21). This system detects both the full length wild type (WT) CXCL8 and the appearance of a neo-epitope (ENWVQ) following SpyCEP cleavage (22). WT CXCL8 was cleaved to completion by SpyCEP in 16 h as observed by loss of the 8.3 kDa substrate (green band) and the appearance of a 6.5 kDa product (red band). No CXCL8 cleavage was observed following incubation with pefabloc-treated SpyCEP or with catalytically dead SpyCEP. Notably, SpyCEP was unable to cleave an N-terminally extended SUMO3-CXCL8 form. However, both C-terminally extended CXCL8 forms, CXCL8-HA and the CXCL8-mucin stalk chimera were cleaved to completion. We subsequently used a sandwich ELISA to quantify chemokine cleavage (Fig. 2*B*), exploiting the fact that the ELISA capture antibody used in multiplex Western blotting loses its ability to bind to CXCL8 following cleavage. ELISA data confirmed our earlier findings, demonstrating near-complete cleavage of WT CXCL8, CXCL8-HA and the CXCL8-mucin stalk chimera within 16 h. N-terminal extension by SUMO3-CXCL8 completely protected CXCL8 from cleavage, with no detected loss of CXCL8 signal. Structural predictions for these extended mutants using AlphaFold2, suggest the cleavage site between Q59- R60 remains exposed (Figs. S1, *A*–*D*, S2, *A*–*E* and S3, *A*–*D*).

### A CXCL8 C-terminal charge reversal is tolerated by SpyCEP

**Figure 3:**
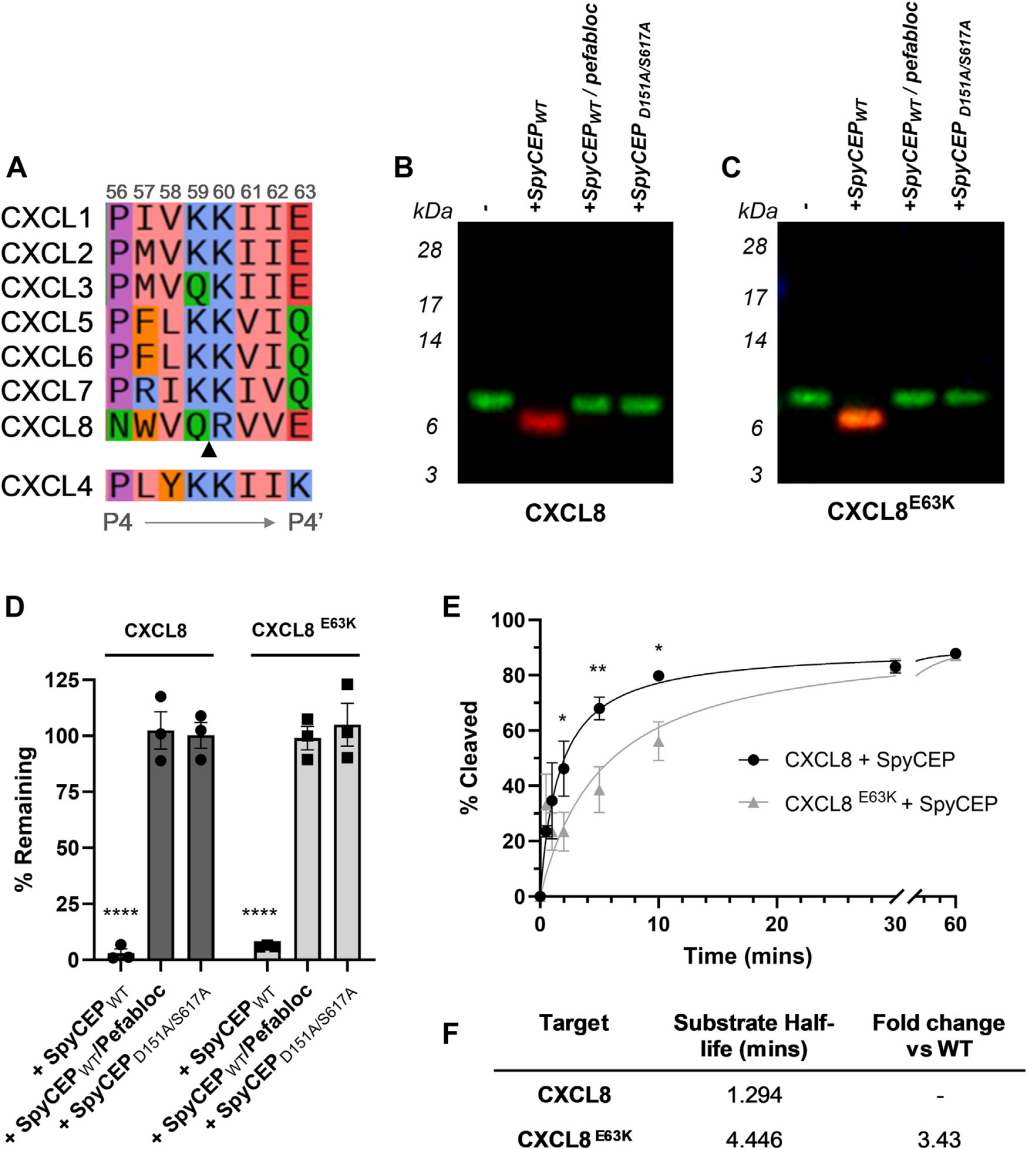
**A charge substitution in the C-terminus of CXCL8 is tolerated by SpyCEP.***A*, Alignment of C-terminal residues within relevant CXC chemokines. The cleavage site within CXCL8 is marked by a *black* triangle. Following incubation of (*B*) WT CXCL8 and (*C*) CXCL8 E63K with SpyCEP for 16 h, cleavage products were imaged by multiplex Western blot. Data are representative of 3 separate experiments. *D*, quantification of chemokine cleavage as determined by ELISA (n = 3). *E*, time course of WT CXCL8 and E63K CXCL8 cleavage by SpyCEP as quantified by ELISA (n = 3). *F*, substrate half-life was calculated from curve fits of data in *panel E*.

Alignment of the C-termini of the known SpyCEP substrates highlighted a K-K pair on either side of the predicted cleavage site in the majority of SpyCEP substrates (Fig. 3*A*), which is partially conserved as Q-R and G-K in CXCL8 and CXCL3 respectively. This corresponds to residues P1-P1′ using standard nomenclature for substrate cleavage sites (23, 24). CXCL4, which is not a SpyCEP substrate, maintains the K-K pairing at position 59 to 60 but has a charge reversal at position P4′, with the E/Q of SpyCEP substrates replaced by K. Since this residue is located within the C-terminal α-helix, we postulated that its basic side chain may explain the resistance of CXCL4 to cleavage by SpyCEP. To test this, we generated a CXCL8 mutant, CXCL8 E63K, replacing the acidic residue with an basic one. Both WT CXCL8 and CXCL8 E63K were cleaved to completion by SpyCEP within 16 h (Fig. 3, *B*–*D*). Time-course assay revealed rapid CXCL8 cleavage by SpyCEP, reaching 80% completion within 10 min (Fig. 3*E*). CXCL8 E63K displayed a significantly reduced cleavage rate, particularly evident within the first 10 min of incubation, although both substrates reached comparable cleavage levels by 60 min. Curve fit analysis reported a 3.43-fold extended half-life for CXCL8 E63K compared to WT CXCL8 (Fig. 3*F*), suggesting that the charge of the residue at the P4′ position plays a relatively minor role in engaging with SpyCEP.

### The N-terminus of CXCL8 confers susceptibility to degradation by SpyCEP

**Figure 4:**
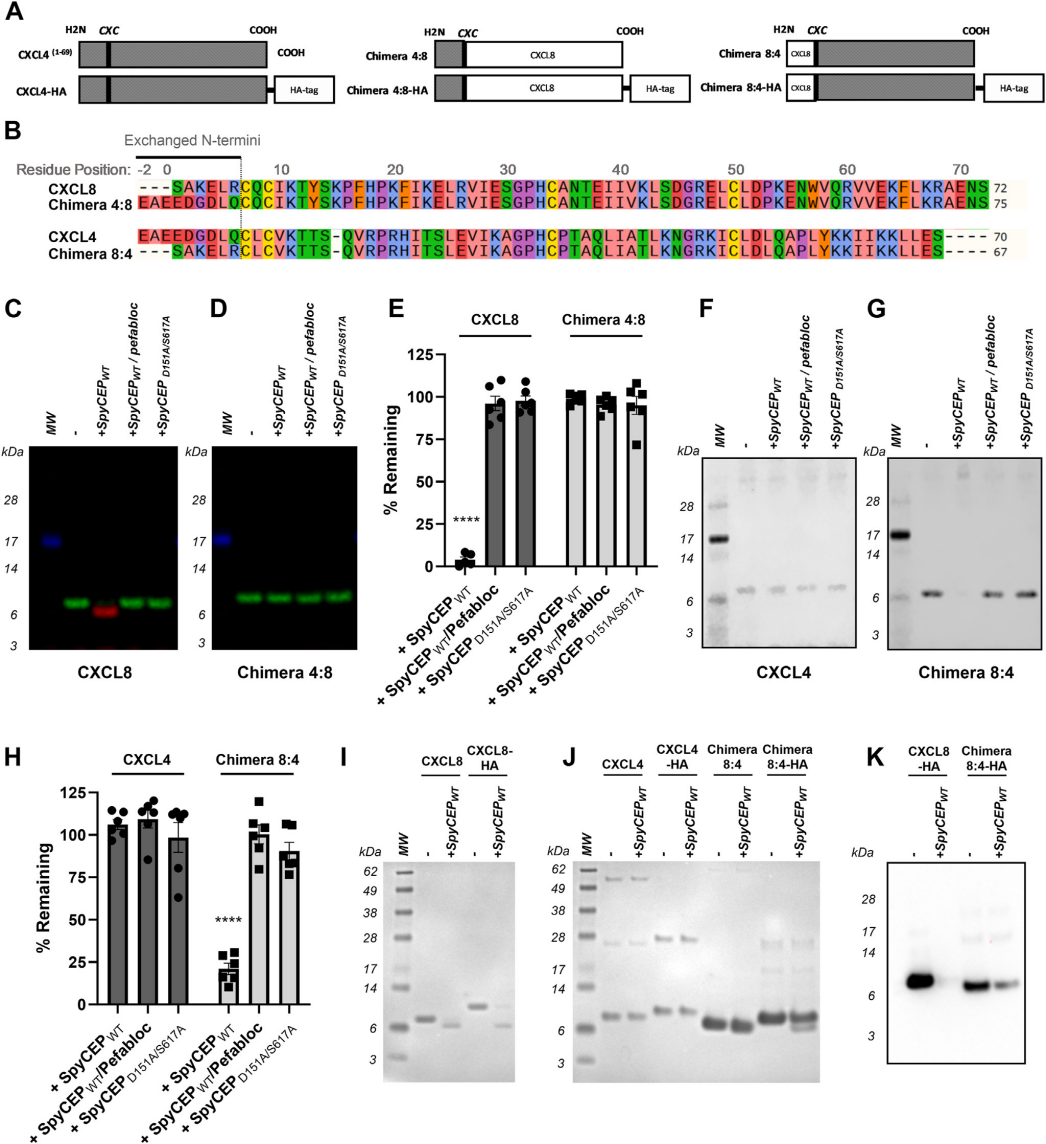
**The N-terminus of CXCL8 is a key determinant of susceptibility to SpyCEP.***A*, cartoon showing CXCL4 and CXCL8-based chimeras with exchanged N-termini, together with the parent molecule CXCL4. *B*, alignment of wild type and chimeric chemokine sequences. *C* and *D*, cleavage products following incubation of WT CXCL8 and chimera 4:8 with SpyCEP for 16 h as imaged by multiplex Western blot (n = 3). *E*, quantification of WT CXCL8 and chimera 4:8 cleavage by CXCL8 ELISA (n = 6). *F* and *G*, cleavage products following incubation of WT CXCL4 and chimera 8:4 with SpyCEP for 16 h, as imaged by monoplex Western blot (n = 3). *H*, quantification of WT CXCL4 and chimera 8:4 by CXCL4 ELISA (n = 6). *I* and *J*, cleavage products following incubation of untagged and HA-tagged variants of WT CXCL8, WT CXCL4, and chimera 8:4 with SpyCEP for 16 h as resolved by SDS-PAGE and imaged by PAGE Blue staining (n = 3). *K*, quantification of CXCL8-HA and chimera 8:4-HA cleavage by Western blot with an anti-HA antibody (n = 3). Western blot data panels are representative of the indicated number of experiments.

Chimeric CXCL8/CXCL4 chemokines were also constructed, with reciprocal exchanges of the N-termini (chimera 4:8 and 8:4, Fig. 4*A*). Chimera 4:8 possesses the first nine residues of CXCL4 (EAEEDGDLQ) and lacks an ELR motif, while chimera 8:4 contains the N-terminal 6 residues (SAKELR) of CXCL8 and is therefore an ELR^+^ chemokine (Fig. 4*B*). Both chemokines were incubated for 16 h with SpyCEP, together with relevant controls. Reaction products were subsequently analyzed by multiplex Western blot. As anticipated, SpyCEP was able to cleave WT CXCL8 (Fig. 4*C*) but was unable to cleave the ELR^-^ chimera 4:8 (loss of function, Fig. 4*D*), findings which were confirmed by ELISA (Fig. 4*E*). In the absence of a neoepitope antibody for CXCL4 cleavage, monoplex Western blotting was used to detect cleavage of CXCL4 and the ELR^+^ chimera 8:4. As observed previously (5), CXCL4 was resistant to SpyCEP cleavage (Fig. 4*F*). In contrast, a gain of function was observed for the ELR^+^ chimera 8:4 (Fig. 4*G*) with a loss of the CXCL4 signal seen following incubation with SpyCEP. These data were corroborated by ELISA, with 16 h incubation sufficient to cleave approximately 80% of chimera 8:4 (Fig. 4*H*).

We assumed that the site of cleavage within chimera 8:4 was likely to be between residues K61-K62 of the CXCL4 sequence which would liberate only a nine amino acid fragment. To better visualize chimera 8:4 cleavage by SDS-PAGE, C-terminally HA-tagged forms of CXCL8, CXCL4, and chimera 8:4 were used. We first confirmed that WT CXCL8 and CXCL8-HA were cleaved to completion by SpyCEP (Fig. 4*I*). Under the same conditions, CXCL4 and CXCL4-HA remained intact, whilst chimera 8:4 and chimera 8:4-HA were both cleaved by SpyCEP, but not to completion (Fig. 4*J*). As anticipated, the size shift from the chimera 8:4-HA variant was predictably larger ∼2 kDa and readily visible. Western blotting and imunodetection with an anti-HA antibody confirmed that CXCL8-HA was cleaved to completion, while approximately 20% of the total signal from chimera 8:4-HA remained (Fig. 4*K*).

### The ELR motif of CXCL8 contributes to efficacious cleavage by SpyCEP

**Figure 5:**
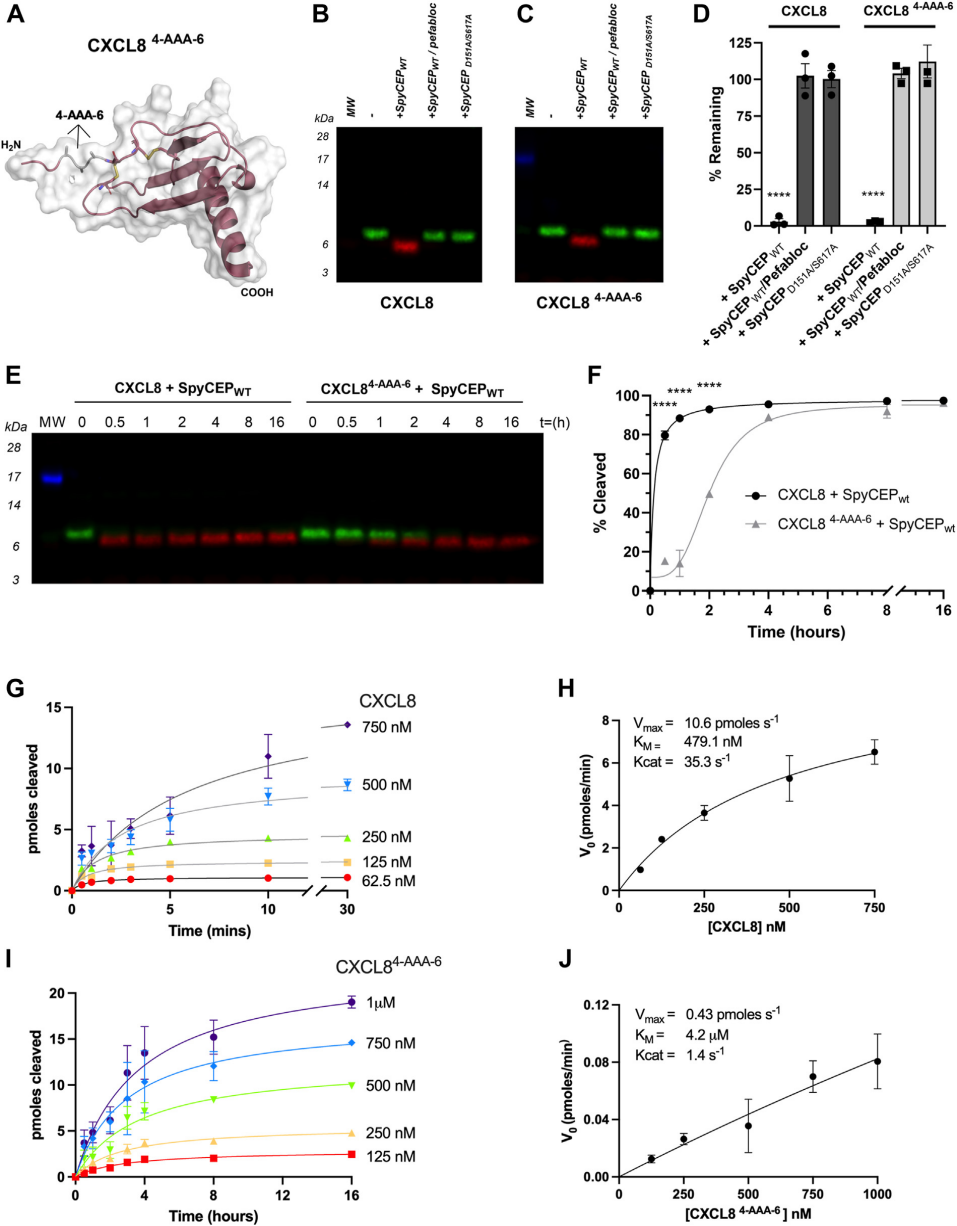
**Substitution of the ELR motif in CXCL8 offers significant protection from SpyCEP cleavage**. *A*, alphaFold2 structural prediction of CXCL8^4-AAA-6^ with mutated residues displayed as sticks. Cleavage products were imaged by multiplex Western blot following incubation of (*B*) WT CXCL8 and (*C*) CXCL8^4-AAA-6^ with SpyCEP for 16 h (n = 3). *D*, quantification of cleavage by CXCL8 ELISA (n = 3). *E*, cleavage products were imaged by multiplex Western blot following incubation of WT CXCL8 and CXCL8^4-AAA-6^ by SpyCEP at regular time intervals (n = 3). *F*, quantification of SpyCEP chemokine cleavage at regular time intervals by CXCL8 ELISA (n = 3). *G–J*, kinetic activity assessment for SpyCEP cleavage of CXCL8 (*G* and *H*), and CXCL8 ^4-AAA-6^ (*I* and *J*) was performed with a fixed concentration of SpyCEP (250 PM), and a range of substrate concentrations. For CXCL8, (*G*) shows pmoles CXCL8 cleaved over 30 min (n = 3), with (*H*) showing the initial velocity of reaction (V_0_) for various [CXCL8] at t = 0.5 min, n = 3). *I*, shows pmoles CXCL8 ^4-AAA-6^ cleaved over 16 h (n = 3), with (*J*) showing V_0_ for various [CXCL8 ^4-AAA-6^] at t = 1 h (n = 3). Kinetic parameters for the cleavage of either substrate are shown inset (*H* and *J*). Data showing the cleavage of WT CXCL8 in *B* and *C* are identical to those shown in Figure 3, *B* and *C*, since these analyses were carried out in parallel.

To directly assess the ELR motif, a CXCL8 mutant was generated (CXCL8 ^4-AAA-6^) in which the motif was replaced by alanine residues (Figs. 5*A* and S4, *A*–*D*) and the sensitivity of the mutant to cleavage by SpyCEP was assessed. Both WT-CXCL8 and CXCL8 ^4-AAA-6^ were cleaved to completion within 16 h (Fig. 5, *B* and *C*) which was confirmed by ELISA (Fig. 5*D*). A time course allowed us to observe the kinetics of cleavage by Western blot (Fig. 5*E*). WT CXCL8 was cleaved completely within 30 min, consistent with prior observations, whilst the CXCL8 ^4-AAA-6^ mutant remained intact after 1 h of incubation with SpyCEP, with cleavage detectable only after 2 h. ELISA corroborated this slower rate of cleavage for the CXCL8 ^4-AAA-6^ mutant, with significant levels of cleavage only observed after 2 h (Fig. 5*F*). Further kinetic analysis of the cleavage of both forms of CXCL8 was undertaken by ELISA, following incubation of a fixed concentration of SpyCEP with various substrate concentrations, [S] (Fig. 5, *G* and *I*). Plots of the initial rates of reaction (V_o_) against [S] (Fig. 5, *H* and *J*) indicated a ∼10-fold increase in the Michaelis constant (K_M_) following mutation (479.1 nM c.f. 4.2 μM), coupled with obvious reductions in the maximal velocity of reaction (V_max_) and turn over number (Kcat). Collectively, these data suggest that the ELR motif is a critical determinant for efficient cleavage of CXCL8 by SpyCEP.

## Discussion

We have previously postulated that the specificity of SpyCEP for ELR + chemokines points to the ELR motif within the substrate N-terminus being a structural requirement for cleavage (5, 18). Here, we provide the first experimental evidence to support that postulate. Akin to a general model of chemokine binding and receptor activation (25, 26, 27) we envisage that chemokine cleavage is a multi-step process requiring an initial substrate binding event prior to cleavage. Given that the ELR motif and cleavage sites are at opposite ends of the chemokine, we surmise that the ELR motif plays a role in the initial tethering of the chemokine to SpyCEP. Although we were unable to directly show a role for the ELR motif in binding in this study, the measurement of K_M_ provides some indication of the apparent affinity of an enzyme for its substrate (28), albeit not a direct measurement of substrate association/dissociation (29). The approximately 10-fold reduction in K_M_ that we observed following the substitution of the ELR motif suggests that the motif likely interacts with SpyCEP. Extension of the CXCL8 N-terminus by incorporating a SUMO-3 domain was also inhibitory, perhaps impeding access of the ELR motif to a binding pocket in SpyCEP. Notably, the inclusion of the ELR motif within the CXCL4 sequence transformed a chemokine that was previously resistant to SpyCEP cleavage, into one that was readily degraded by SpyCEP.

In humans, CXCL8 is produced as two variants of 72 and 77 amino acids which vary only by the length of their N-termini. Both isoforms contain an ELR motif and are readily cleaved by SpyCEP (5), indicating tolerance for minor N-terminal extensions within the substrate binding pocket, but not the larger SUMO-3 extension. In contrast, a variety of C-terminal CXCL8 extensions were tolerated by the enzyme, ranging from a relatively small HA-tag (1.102 kDa) to a much larger mucin-like stalk (34.8 kDa). This suggests that CXCL8 enters the SpyCEP binding pocket with the N-terminal end first and the extreme C-terminus of the chemokine accessible to the solution. Interestingly, the sole mutation at the P4′ position within the chemokine C-terminus (CXCL8 E63K) produced a substrate that could be cleaved by SpyCEP into a fragment of identical size indistinguishable from WT CXCL8 in terms of apparent molecular weight and immunoreactivity with a neo-epitope antiserum. This suggests that cleavage of CXCL8 E63K also occurs between Q59-R60 and that the extreme C-terminus of CXCL8 is unlikely to form highly specific interactions with SpyCEP. In recent reports, SpyCEP was shown to tolerate major reorganizations of the catalytic triad, retaining catalytic activity even when lacking a critical residue D115 (21). It is plausible that some apparent redundancy in the enzyme catalytic site contributes to the broad targeting of sequentially distinct, yet structurally homologous substrates by SpyCEP.

Our work supports a broader effort aimed at understanding the structural basis for substrate recognition by SpyCEP, with the aim of rational design of vaccines protecting against *S. pyogenes* infections (30, 31, 32, 33, 34, 35). In mice, antibodies raised against SpyCEP following vaccination typically exert their protective effects by inhibiting enzyme activity rather than by opsonization (36). Recent work by Pearson *et al.,* (21) lends support to including the SpyCEP C-terminal binding domain in a candidate vaccine, as a refinement of the earlier ‘CEP5’ based vaccines that spanned SpyCEP residues 35 to 587, encompassing the enzyme N-terminus and part of the C-terminus (37). Based on our findings reported here, identification of the SpyCEP domain that interacts with the ELR motif of CXCL8 may provide useful epitopes against which blocking antibodies may be developed. Key to the identification of these epitopes will be the structural determination of enzyme:substrate complexes to complement the existing high-resolution SpyCEP structures (16, 17).

## Experimental procedures

### Materials

Chemicals were from Sigma. Oligonucleotides were from MWG-Biotech. HPLC materials were from Cytiva. Recombinant CXCL8-mucin stalk chimera (38) was purchased from R&D Systems. Recombinant SpyCEP was produced by Genscript as previously described (21).

### *In silico* analyses

Multiple sequence alignments were performed using CLUSTAL-W within SnapGene software (www.snapgene.com). Protein structure predictions were generated with AlphaFold2 (DeepMind; EMBL-EBI) using the ColabFold interface (39, 40, 41), with 12 iterances of model recycling. The PyMOL Molecular Graphics System, Version 3.0 (Schrödinger, LLC) was used to visualize solved/predicted structures of chemokines.

### Production of recombinant proteins

The pE-SUMOpro3 AMP vector (Lifesensors) was modified by the inclusion of an *AgeI* site for chemokine subcloning as previously described (42). ORFs encoding WT CXCL8 (aa 27–99), and WT CXCL4 (aa 32–101) were subcloned at *AgeI* and *BamHI* sites of the vector. cDNAs encoding chimeric chemokines and HA-tagged variants were generated by overlap extension PCR prior to ligation into pE-SUMOpro3. Site-directed mutagenesis of CXCL8 was performed with QuikChange II Site-directed mutagenesis kit (Agilent Technologies). The authenticity of all inserts was verified by Sanger sequencing of plasmid DNA by MWG-Biotech.

CXCL4-based proteins were expressed from inclusion bodies in BL21(DE3) PLysS *E.coli* and purified as described previously (43). For CXCL8-based proteins, expression and purification were carried out in Shuffle T7 Competent *E.coli* as described previously (19). Removal of the SUMO3 tag was achieved by incubation with *Ulp1* (42). Refolding of CXCL4-based proteins was performed by infinite dilution in cystine-cysteine refolding buffer (44). Chemokines were concentrated by heparin affinity chromatography. SDS-PAGE and Western blot confirmed protein identity and purity. Chemokine concentration was assessed by semi-quantitative SDS-PAGE, BCA assay, and CXCL4/CXCL8 ELISA.

### Chemokine cleavage

Chemokine cleavage was assessed as previously described (21). Briefly, chemokines and SpyCEP were incubated for 16 h assays at 37 °C in a final volume of 20 μl PBS (pH 7.4) using a 1:1000 enzyme: substrate ratio. PBS was supplemented with 0.1% BSA where appropriate. Control digests lacked SpyCEP, contained SpyCEP pre-incubated with Pefabloc (2 mg/ml), or the catalytically inactive D151A/S617A SpyCEP. Reactions were terminated by adding 100 mM DTT, 4× NuPage LDS sample buffer, and heating to 70°C for 5 min (SDS-PAGE) or adding 2 mg/ml pefabloc (ELISA) before analysis.

### SDS-PAGE/Western blot

Proteins were resolved by reducing SDS-PAGE on 10% NuPAGE Bis-Tris gels and visualized by PAGE-blue staining. Western blots were performed using iBlot transfer with nitrocellulose membranes blocked in PBS-T with 5% milk. Membranes were probed with 0.1 μg/ml primary antibodies overnight at 4 °C. Multiplex Western blots included CXCL8 detection with anti-CXCL8 mAb (MAB208, R&D Systems) and 1:300 anti-ENWVQ antisera (in-house) as described in (21). For the detection of CXCL4 and chimera 8:4, anti-CXCL4 mAb (MAB7951) was used. Secondary antibodies (anti-mouse; A11357/rabbit; A21109 Alexa Fluor conjugates) were used at a concentration of 0.01 μg/ml and detected with an Odyssey XF imager. HA-tagged proteins were detected with anti-HA-HRP mAb (Roche) and imaged with ECL substrate (Pierce) using an iBright instrument.

### CXCL8 and CXCL4 sandwich ELISA

SpyCEP catalytic activity was measured by detecting intact CXCL8 or CXCL4 using CXCL8/CXCL4 DuoSet ELISA kits (DY208/DY795, R&D Systems) according to the manufacturer's instructions as previously described (21). Substrate half-life was calculated from time-course data by curve-fitting to a non-linear hyperbola using Prism 9.2 (GraphPad).

### Kinetic activity assessment of SpyCEP

Kinetic activity assessment was performed over assay-dependent time courses, where a fixed concentration of recombinant SpyCEP 250 PM, was incubated for the indicated times with various concentrations of CXCL8, or CXCL8 ^4-AAA-6^, at 37 °C in a final volume of 200 μl PBS +0.1% BSA. Reactions were terminated by the addition of 2 μl of 10 mg/ml pefabloc to 20 μl of the sample, and diluted for analysis by CXCL8 DuoSet ELISA kit (DY208, R&D Systems). The kinetic activity was assessed by calculating the number of pmoles cleaved over time, and plotting the initial rate of reaction in pmoles cleaved/minute.

### Statistical analyses

All data are presented as mean ± SEM. Statistical analyses were performed in Prism 9.2, using 2-way ANOVA with multiple comparisons, followed by Dunnett’s post-test. For time courses, Sidak’s post-test was used. Statistical significance is presented as ∗*p* < 0.05, ∗∗*p* < 0.01, ∗∗∗*p* < 0.001 and ∗∗∗∗*p* < 0.0001.

## Data availability

The data that support the findings of this study are available from the corresponding author upon request.

## Supporting information

This article contains supporting information.

## Conflict of interest

The authors declare that they have no conflicts of interest with the contents of this article.
