# Supplementary material for: The N-terminal ELR+ motif of the neutrophil attractant CXCL8 confers susceptibility to degradation by the Group A streptococcal protease, SpyCEP
Source: J Biol Chem. 2025 Mar 25;301(5):108448. doi: 10.1016/j.jbc.2025.108448 (PMC12022482; doi:10.1016/j.jbc.2025.108448)
Supplement: Supporting information [file mmc1.docx]

**Supporting information: The N-terminal ELR^+^ motif of the neutrophil attractant CXCL8 confers susceptibility to degradation by the Group A Streptococcal protease, SpyCEP**

Sean Patrick Giblin^1^, Sophie McKenna^2^, Stephen Matthews^2,3^, Shiranee Sriskandan^3,4^ and James Edward Pease^1^.s

^1^National Heart and Lung Institute, Imperial College London, London, United Kingdom.

^2^Department of Life Sciences, Imperial College London, London, United Kingdom.

^3^Centre for Bacterial Resistance Biology, Imperial College London, London, United Kingdom.

^4^Department of Infectious Disease, Imperial College London, London, United Kingdom.

Keywords: chemokine, CXCL8, enzyme degradation, protease, recombinant protein expression, SpyCEP, Streptococcus pyogenes (S. pyogenes)

Running title: The ELR motif of CXCL8 is required for processing by SpyCEP

Corresponding author: James Pease, [j.pease@imperial.ac.uk](mailto:j.pease@imperial.ac.uk)


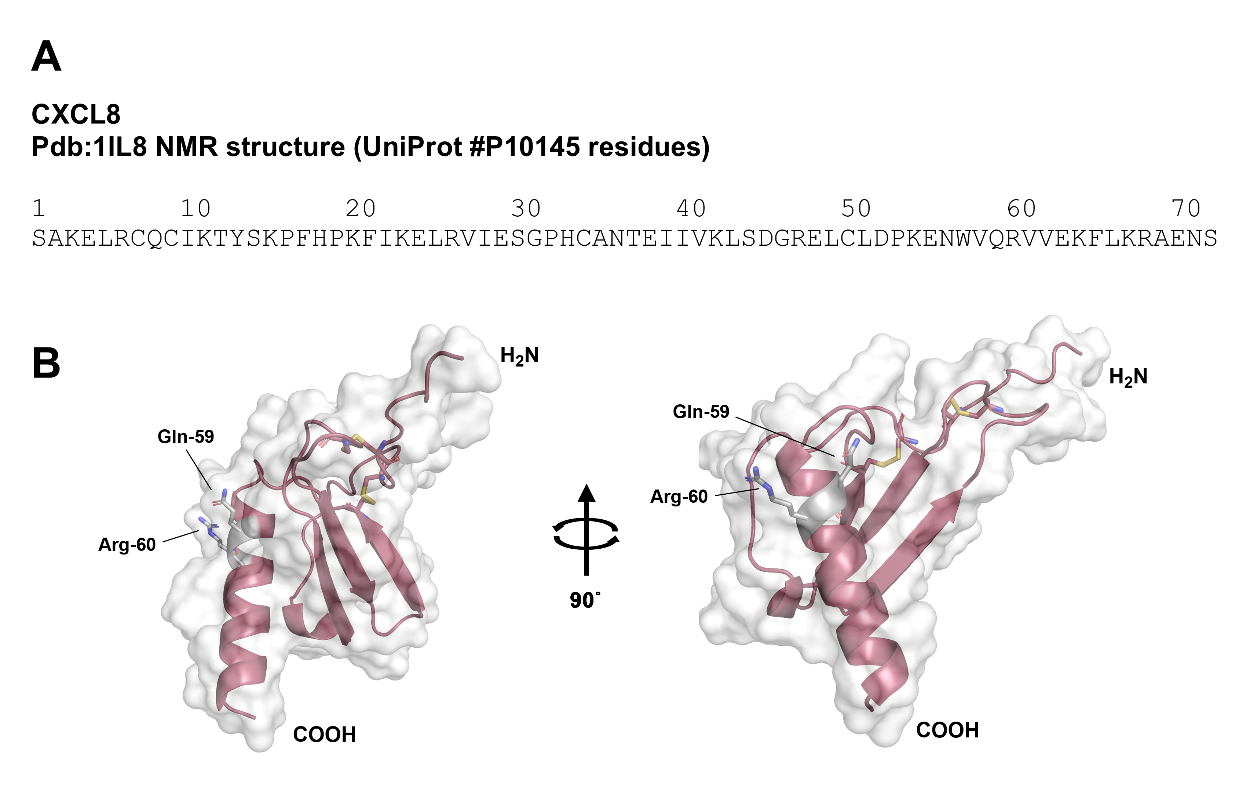


**Figure S1. Protein structure of CXCL8 with the known SpyCEP cleavage site Gln-59/Arg-60 indicated.** (**A**) The amino acid sequence of CXCL8 corresponding to the NMR structure (pdb:1IL8). (**B**) The structure of CXCL8 with the known SpyCEP cleavage site at Gln-59/Arg-60 indicated; with 90˚ *Y*-axis rotation.

**
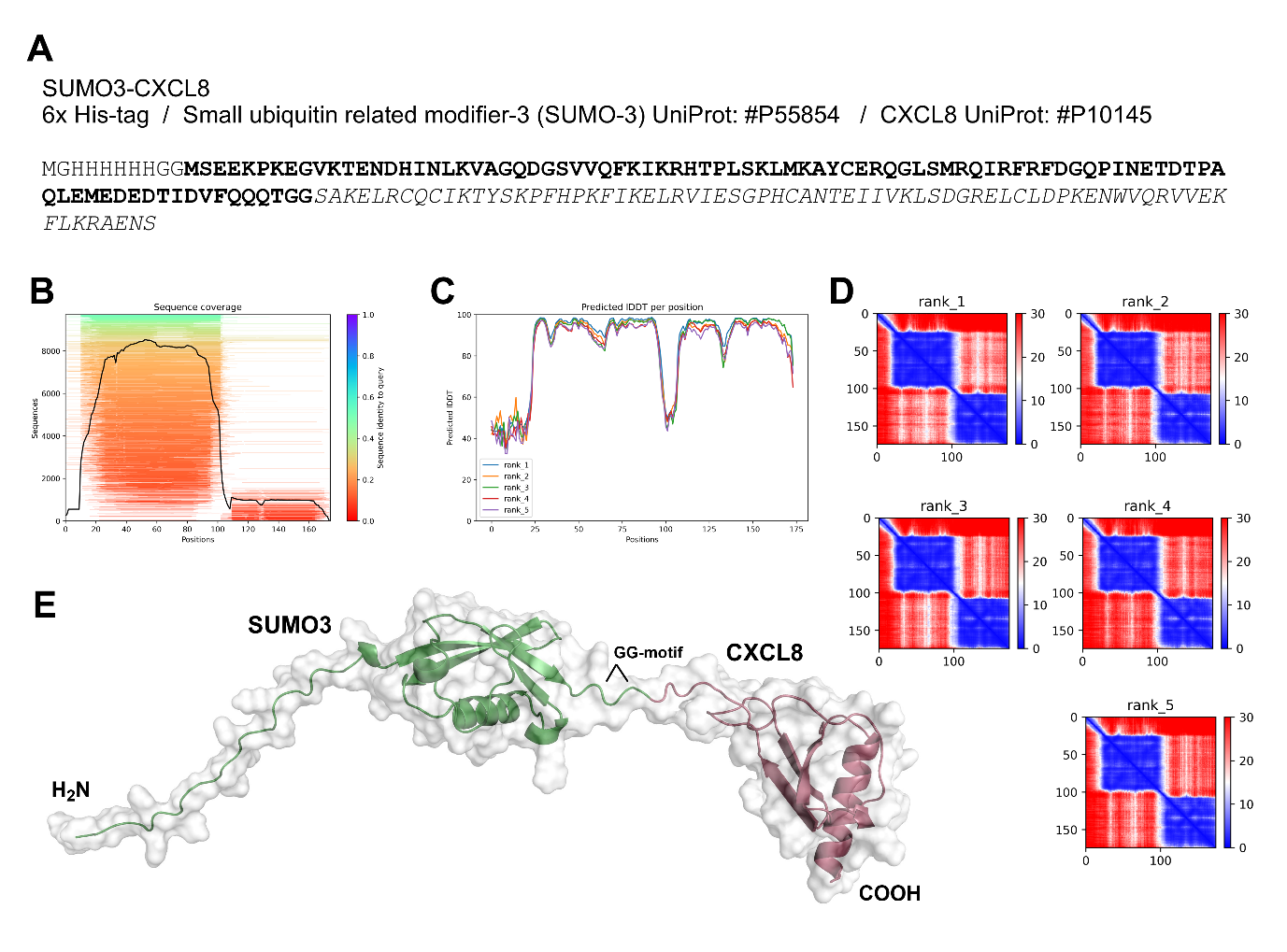
**

**Figure S2. Structure prediction of SUMO3-CXCL8.** (**A**) Displays the amino acid sequence for SUMO3-CXCL8. The structure of SUMO3-CXCL8 was predicted by AlphaFold2, with (**B**) Sequence coverage, (**C**) plDDT scores and (**D**) Plots of PAE per residue position for the top 5 ranking models displayed. (**E**) The structure of the top-ranking model. Residues corresponding to CXCL8 are dark red, and the N-terminal extension is dark green.

**
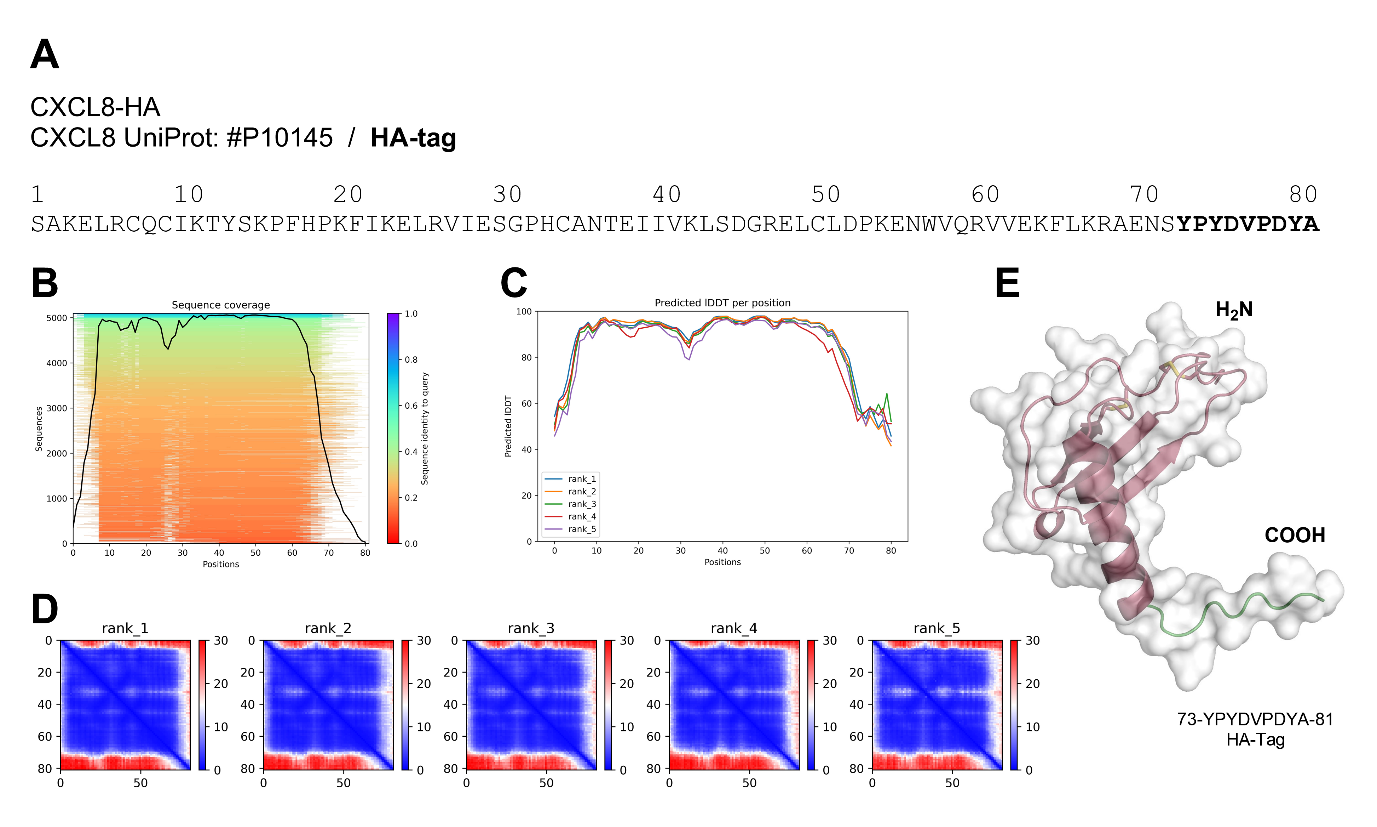
Figure S3. Structure prediction of CXCL8-HA.** (**A**) Displays the amino acid sequence for CXCL8-HA. The structure of CXCL8-HA was predicted by AlphaFold2, and (**B**) Sequence coverage, (**C**) plDDT scores and (**D**) Plots of PAE per residue position for the top 5 ranking models are presented. (**E**) The structure of the top-ranking model. Residues corresponding to CXCL8 are colored red, and the C-terminal extension is green.

**
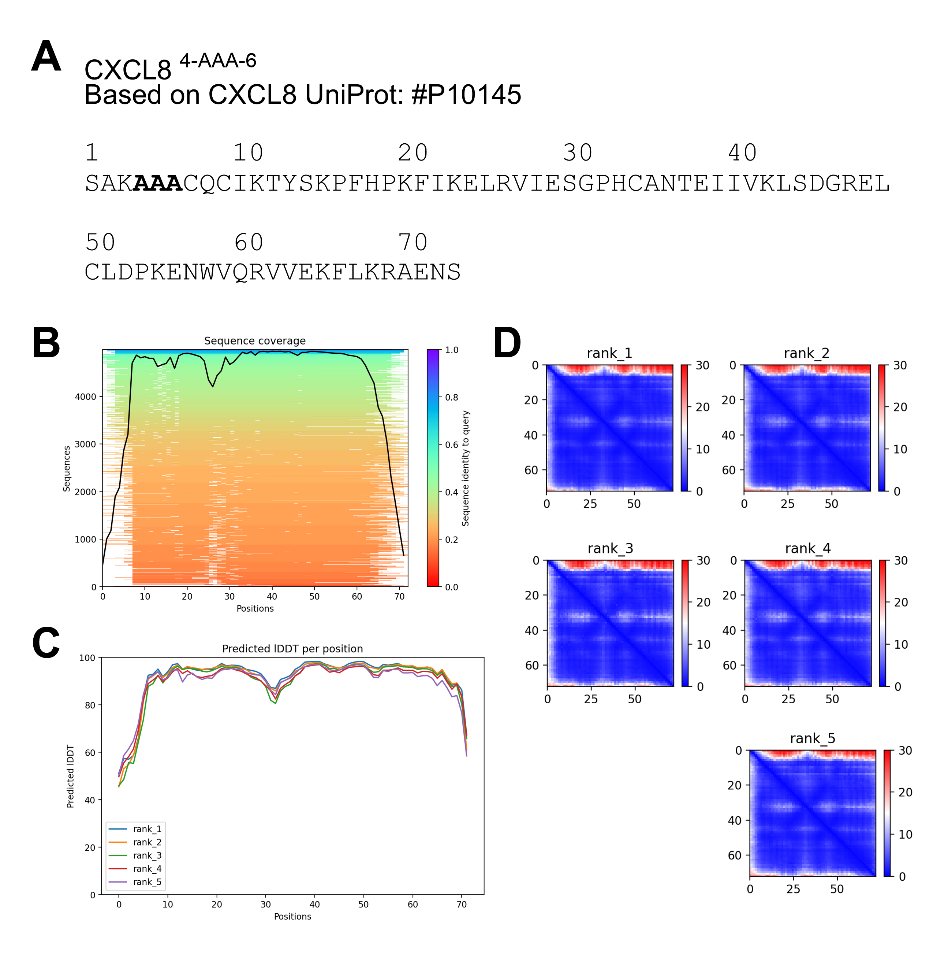
**

**Figure S4. Structure prediction of CXCL8 ^4-AAA-6^.** (**A**) The amino acid sequence for CXCL8 ^4-AAA-6^. The structure of CXCL8 ^4-AAA-6^ was predicted by AlphaFold2 and the (**B**) Sequence coverage, (**C**) plDDT scores and (**D**) Plots of PAE per residue position for the top 5 ranking models are displayed. The structure of the top-ranking model is presented in Figure 5A.
